# Real-Time AI-Augmented Fluoroscopic Navigation for Intraoperative Pulmonary Nodule Localization: Prospective Observational Pilot Study

**DOI:** 10.2196/94084

**Published:** 2026-09-09

**Authors:** Hsiang Teng, Hsu-Kai Huang, Cheng-Jung Lin, Ying-Shian Chen, Yueh-Hsun Tsai, Tsai-Wang Huang, Kuan-Hsun Lin

**Affiliations:** 1 Division of Thoracic Surgery, Department of Surgery, Tri-Service General Hospital National Defense Medical University Taipei Taiwan; 2 Department of Thoracic Surgery, School of Medicine, College of Medicine National Defense Medical University Taipei Taiwan; 3 Kaohsiung Armed Forces General Hospital Kaohsiung Taiwan; 4 Taichung Armed Forces General Hospital Taichung Taiwan; 5 Digital Medical Center, Tri-Service General Hospital National Defense Medical University Taipei Taiwan

**Keywords:** artificial intelligence, AI, augmented fluoroscopy, intraoperative navigation, computer-assisted surgery, digital surgical planning, pulmonary nodule localization

## Abstract

**Background:**

The integration of AI into intraoperative surgical imaging represents an emerging frontier in digital health. Despite advances in preoperative computed tomography (CT)–based surgical planning, real-time translation of imaging data into actionable intraoperative guidance remains limited by CT-to-body divergence—a fundamental information gap between preoperative digital models and the dynamic surgical field. This divergence, driven by lung deflation under anesthesia and positional changes, represents a critical digital-to-physical registration challenge that current preoperative imaging workflows fail to address in real time.

**Objective:**

This study aimed to evaluate the clinical feasibility, localization success, and safety of the LungVision system—an AI-augmented fluoroscopic navigation platform—for real-time intraoperative localization of small pulmonary nodules during thoracoscopic surgery.

**Methods:**

A prospective single-center study enrolled 14 patients with pulmonary nodules requiring localization prior to thoracoscopic resection between January 2024 and December 2024. The platform comprises a passive radiopaque positioning board, an AI-powered computing unit for real-time image processing, and a tablet-based interface for procedural planning and augmented visualization. All patients received dual localization with either preoperative CT-guided dye injection or Archimedes virtual bronchoscopic navigation followed by intraoperative localization with the LungVision system and video-assisted thoracoscopic surgery. Demographic data, lesion characteristics, procedural performance, and procedure-related complications were recorded.

**Results:**

The mean patient age was 57.2 (SD 9.2) years, and 92.9% (13/14) were nonsmokers. Most nodules were peripherally located (12/14, 85.7%), with a mean diameter of 9.3 (SD 5.3) mm and a mean CT attenuation of −320.1 (SD 334.9) Hounsfield units. LungVision successfully localized all target lesions intraoperatively, with a mean navigation time of 38.6 (SD 19.5) minutes. Complete resection was achieved in all cases, and 71.4% (10/14) of nodules were pathologically malignant. No intraoperative or localization-related complications were observed. The system was integrated into the existing operating room without additional infrastructure modifications.

**Conclusions:**

In this prospective study, the LungVision system achieved successful intraoperative localization of small, hypodense pulmonary nodules using a bronchoscopic approach integrated with conventional C-arm fluoroscopy. These findings support the feasibility of the technique and provide preliminary evidence on its use in thoracoscopic resection workflows. Larger studies are needed to further evaluate its clinical performance and implementation in diverse practice settings.

**Trial Registration:**

ClinicalTrials.gov NCT07682012; https://clinicaltrials.gov/study/NCT07682012

## Introduction

With the widespread implementation of lung cancer screening programs and growing public awareness, an increasing number of individuals are benefiting from low-dose computed tomography (CT) screening [1]. Several randomized trials have demonstrated that low-dose CT screening can significantly reduce lung cancer–related mortality among high-risk populations [2]. Consequently, the enhanced sensitivity of early detection has led to a notable increase in surgical management for small, subsolid, or ground-glass opacity (GGO) pulmonary nodules, many of which are deeply embedded within the lung parenchyma [3]. These lesions are often difficult to visualize or palpate during video-assisted thoracoscopic surgery (VATS), making accurate intraoperative localization essential to ensure complete resection while preserving normal lung tissue.

The application of AI to intraoperative surgical guidance has gained momentum as a key area of digital health innovation [4,5]. Computer-assisted surgery platforms ranging from robotic systems to AI-enhanced imaging tools aim to bridge the gap between preoperative planning data and real-time surgical execution [6]. However, despite rapid advances in AI-driven surgical technologies, only a small proportion of digital health applications have been directly implemented in the operative setting [5], and few AI-assisted surgical navigation systems have been deployed in real-world clinical practice [7]. Existing solutions such as robotic bronchoscopy platforms (eg, the Ion and MONARCH platforms) require significant capital investment and dedicated infrastructure. There is a growing need for portable, software-based AI platforms that can augment existing operating room equipment—particularly conventional C-arm fluoroscopy—without requiring hybrid operating room setups or proprietary robotic hardware [8]. CT-to-body divergence has been reported in prior studies to be clinically relevant in bronchoscopic navigation, with reported values varying across imaging platforms and procedural conditions [9]. Moreover, conventional CT-guided preoperative localization techniques carry substantial complication rates, including pneumothorax (35%) and marker dislodgment (2.4%-13%) [10]. These limitations highlight the need for real-time, AI-assisted intraoperative solutions that can dynamically compensate for positional changes.

To address these limitations and reduce CT-to-body divergence, our institution evaluated the LungVision system (Body Vision Medical). This software-based digital navigation platform integrates preoperative CT datasets with real-time C-arm fluoroscopy through AI-driven image fusion. Notably, the system operates as an add-on to conventional C-arm fluoroscopy and does not require proprietary hardware, electromagnetic field generators, or hybrid operating room infrastructure. This multimodal approach provides augmented fluoroscopic navigation intended to support bronchoscopic guidance and path planning. The present study represents, to our knowledge, one of the first prospective clinical evaluations of the LungVision system for intraoperative localization of pulmonary nodules prior to surgical resection and one of the first reported clinical applications in Asia. Given the exploratory nature and limited sample size of this pilot study, the analysis was designed to assess feasibility and safety rather than establish formal noninferiority. The aim was to assess the feasibility, localization success, and safety of this AI-driven platform for intraoperative localization of small pulmonary nodules.

## Methods

### Patient Enrollment

This was a prospective, single-arm, single-center observational pilot study. Patients were enrolled consecutively between January 2024 and December 2024 at the Division of Thoracic Surgery, Department of Surgery, Tri-Service General Hospital, Taipei, Taiwan. Eligible patients were adults aged 18 to 65 years scheduled to undergo thoracoscopic resection for solitary, subsolid, or ground-glass nodules requiring localization based on lesion characteristics and CT imaging as determined by the attending surgeon. All participants were required to be able and willing to complete the study protocol.

No formal sample size calculation was performed because the study was exploratory in nature. The planned enrollment target was 20 patients, determined pragmatically based on expected patient availability and available study resources. During data verification, 30% (n=6) of the enrolled patients were identified as not meeting the institutional review board (IRB)–approved age eligibility criterion (18-65 years) and were therefore excluded from the final analysis according to the study protocol, resulting in a final analytic cohort of 14 patients.

### Ethical Considerations

This study was conducted in accordance with the principles of the Declaration of Helsinki and was approved by the IRB of the Tri-Service General Hospital, Taipei, Taiwan (A202303004). This study was registered on ClinicalTrials.gov (NCT07682012; retrospectively registered on July 2, 2026); the study protocol, primary outcome definition, and eligibility criteria were prespecified in the IRB-approved protocol (version 6.0, dated June 3, 2024) before any data collection, and no outcome measures were modified after enrollment began. Written informed consent was obtained from all participants prior to enrollment and before any study-related procedures were performed. Participants consented to the use of their anonymized clinical data for research and publication purposes. All study data were deidentified before analysis and stored in a secure institutional database accessible only to authorized study personnel. No financial compensation was provided to study participants.

### System Architecture and AI Processing Pipeline

#### Hardware Components

**Figure 1:**
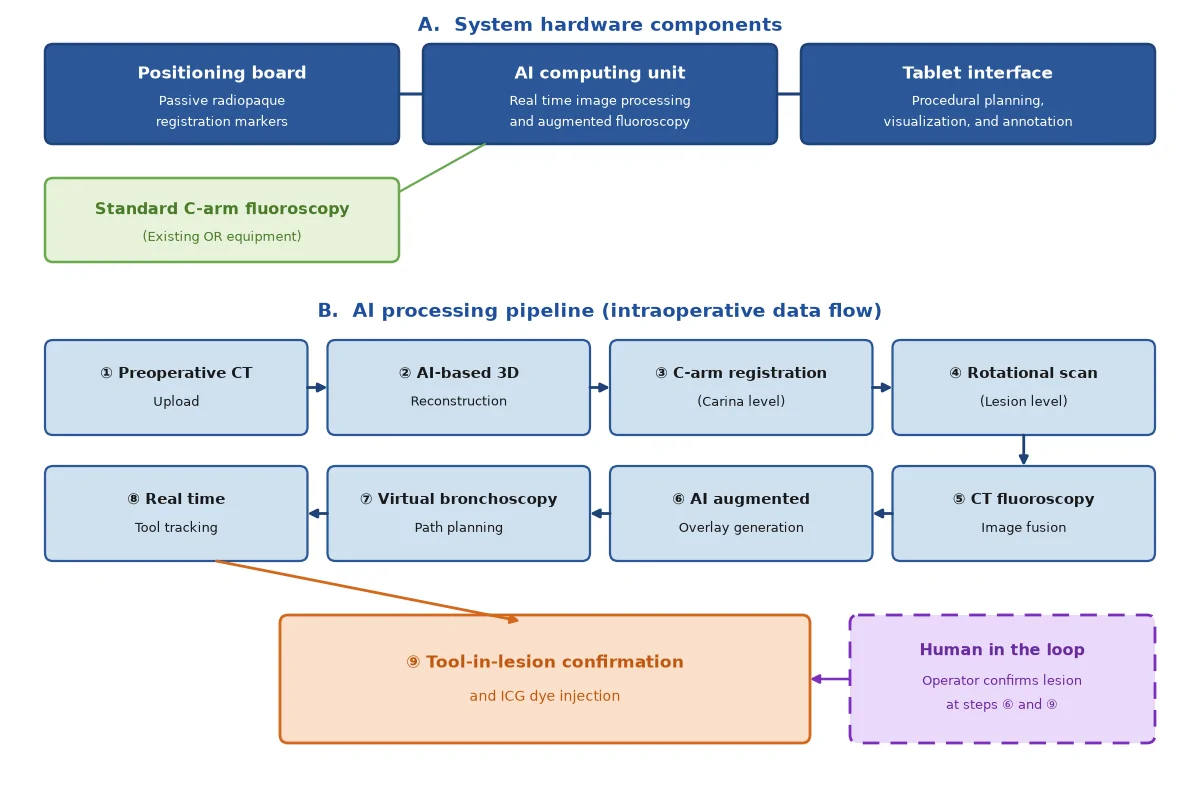
System architecture and AI processing pipeline of the LungVision platform. (A) The system comprises 3 hardware components (positioning board, AI computing unit, and tablet interface) integrated with existing C-arm fluoroscopy infrastructure. (B) The data pipeline illustrates the sequential AI-driven stages from preoperative computed tomography (CT) processing to real-time intraoperative navigation to lesion confirmation and marking. ICG: indocyanine green; OR: operating room.

The LungVision system comprises 3 primary components (Figure 1A). The first is the patient positioning board—a passive, mobile platform embedded with radiopaque bead layers for anatomical registration; it contains no electromagnetic elements or wired components. The second is the main computing unit responsible for AI-based image processing, procedural planning, and real-time augmented fluoroscopy. The third is a tablet interface supporting case planning; virtual bronchoscopy; real-time adjustments; lesion annotation; and intraoperative visualization, including digital zoom and lesion boundary display.

#### AI Processing Pipeline

The LungVision system integrates real-time C-arm fluoroscopy with AI-based image-processing algorithms and augmented fluoroscopic visualization to support intraoperative navigation toward pulmonary nodules that are often difficult to detect using conventional imaging modalities (Figure 1B).

### Imaging Acquisition Requirements

Preoperative CT scans were obtained in the supine position at end inspiration with or without contrast enhancement using the following imaging parameters: slice thickness of 1.5 mm or less, slice spacing of 2 to 3 mm with 20% to 50% overlap, pixel resolution of 0.418 to 0.824 mm, and reconstruction matrix of 512 × 512.

### LungVision System Localization Workflow

The LungVision processing pipeline proceeds sequentially from preoperative CT upload and AI-based 3D reconstruction to intraoperative C-arm registration at the level of the main carina and a rotational scan centered on the lesion to CT-to-fluoroscopy image fusion, AI-generated augmented fluoroscopic overlay, virtual bronchoscopic path planning, and continuous real-time tool tracking, culminating in tool-in-lesion confirmation and targeted indocyanine green (ICG) dye injection (Figure 1B).

**Figure 2:**
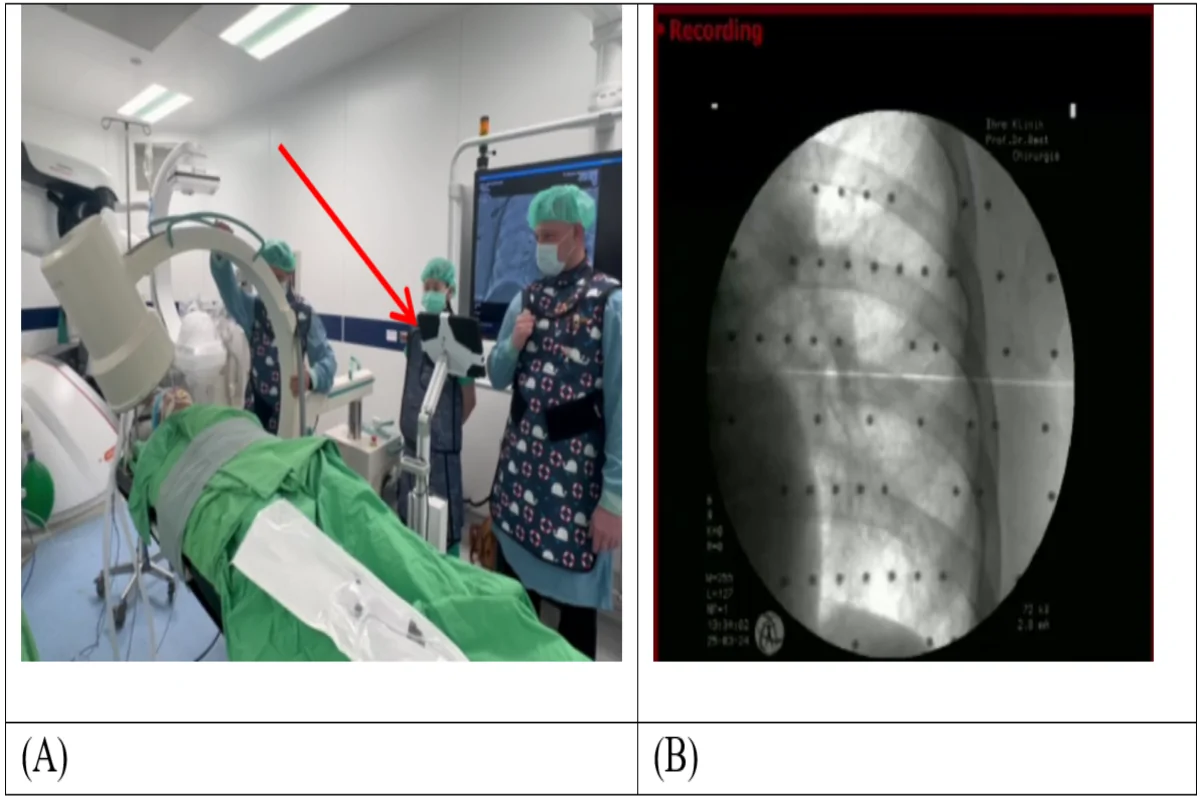
Augmented fluoroscopic localization using the LungVision system. (A) The patient is positioned on a dedicated reference board embedded with radiopaque markers. The LungVision main unit processes intraoperative computed tomography (CT) images acquired by the C-arm and displays navigation guidance via the tablet interface (red arrow). (B) During C-arm rotation, real-time imaging captures a sequence focused on the lesion, which is visualized on the tablet. This AI-enhanced system reconstructs 3D anatomy from standard 2D fluoroscopy to support intraoperative localization by reducing the impact of CT-to-body divergence.

After system initialization and uploading of the preoperative CT scan, the LungVision platform (Figure 2A) commences the localization process with a critical step: rotational C-arm fluoroscopy at the level of the main carina. This step enables real-time anatomical registration by aligning intraoperative fluoroscopic images with the preoperative CT dataset. The system subsequently performs a comparative analysis to detect positional discrepancies and provides suggestions via the tablet interface for optimal C-arm adjustment toward the target lesion.

**Figure 3:**
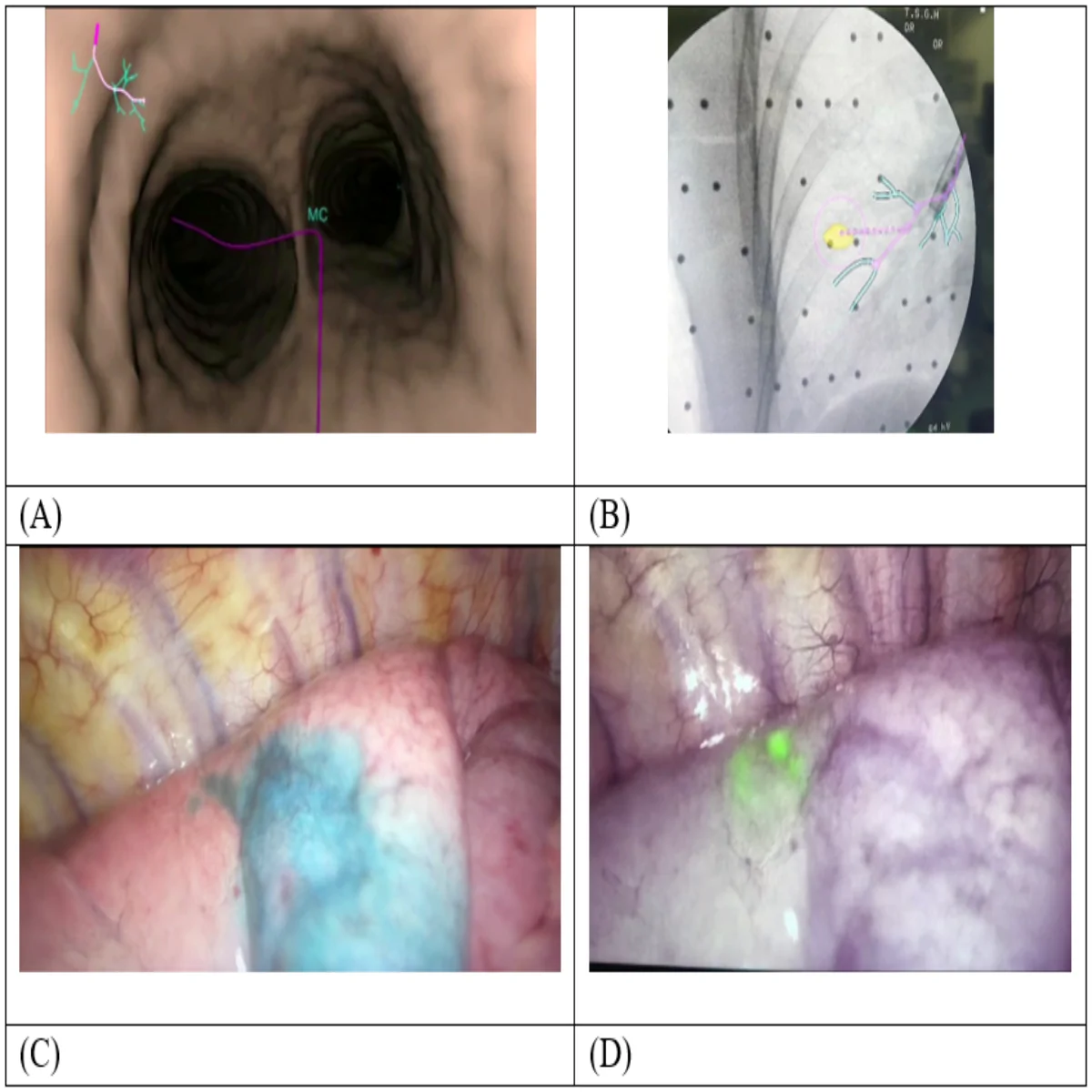
Intraoperative navigation and dual-dye confirmation of the target lesion. (A) A virtual bronchoscopy route was generated after image processing, guiding navigation toward the target lesion. (B) Augmented fluoroscopy with a superimposed overlay of the lesion enabled real-time, image-guided navigation and tool-in-lesion confirmation. (C) Intraoperatively, the blue dye injected during preoperative computed tomography–guided localization was visualized during video-assisted thoracoscopic surgery. (D) Upon switching to near-infrared imaging, the indocyanine green fluorescence corresponded to the same region, confirming accurate localization.

Subsequently, a second rotational scan centered on the lesion is then performed to refine spatial alignment and visualize surrounding anatomical structures (Figure 2B). At this stage, the operator can manually identify the lesion on the fluoroscopic overlay to update and confirm its intraoperative location. On the basis of this updated confirmation, the LungVision system generates an optimized virtual bronchoscopic pathway to the target lesion (Figure 3A), accounting for anatomical complexity and lesion depth. For peripheral lesions, the system offers precise guidance regarding navigation direction and distance.

Throughout the procedure, standard C-arm fluoroscopy is augmented with a real-time overlay, enabling continuous visualization of the bronchoscope and the associated instruments relative to the lesion (Figure 3B). Once the target is reached, localization is completed by injecting ICG dye through an extended catheter to mark the lesion for subsequent surgical resection (Multimedia Appendix 1).

### Localization and Surgical Procedure

Preoperative tumor localization was performed using methylene blue injection via either CT-guided percutaneous technique or the Broncus Medical Archimedes virtual bronchoscopic navigation system (Figure 3C). Subsequently, all patients underwent intraoperative localization in the operating room with the LungVision system, using C-arm–based tomography to acquire volumetric imaging data, enabling real-time AI-driven reconstruction, and delivering ICG dye to the target lesion site for tumor marking (Figure 3D). VATS with tumor resection was then performed by experienced thoracic surgeons from the Department of Thoracic Surgery at Tri-Service General Hospital.

### Evaluation

The primary outcome of this study was the localization success rate, defined as the proportion of patients in whom fluoroscopic tool-in-lesion confirmation was achieved using the LungVision system. More specifically, the navigation catheter was confirmed by the operating surgeon to be positioned within the target nodule boundary as visualized on the real-time LungVision fluoroscopic overlay prior to ICG injection. Secondary outcomes included complete resection rate, navigation time (from system initialization to tool-in-lesion confirmation), total surgical duration, and the incidence of perioperative complications. These outcome measures were selected to assess the clinical feasibility, localization success, and safety of the LungVision system for intraoperative tumor localization in thoracoscopic lung resections.

### Statistical Analyses

Continuous variables were summarized as means with SDs, whereas categorical variables were presented as frequencies and percentages. Given the single-arm, descriptive design of this pilot study, no formal hypothesis testing was performed. Descriptive and clinical statistical analyses were conducted using the SPSS software (version 26.0; IBM Corp). Figures were generated using Python (version 3.12; Python Software Foundation) with *matplotlib* (version 3.10.7) and seaborn (version 0.13.2). In addition, an exploratory ordinary least squares linear regression of navigation time on case sequence number was performed using Python to examine whether procedural time changed over successive cases; this analysis was not prespecified and is reported in Multimedia Appendix 2.

## Results

**Figure 4:**
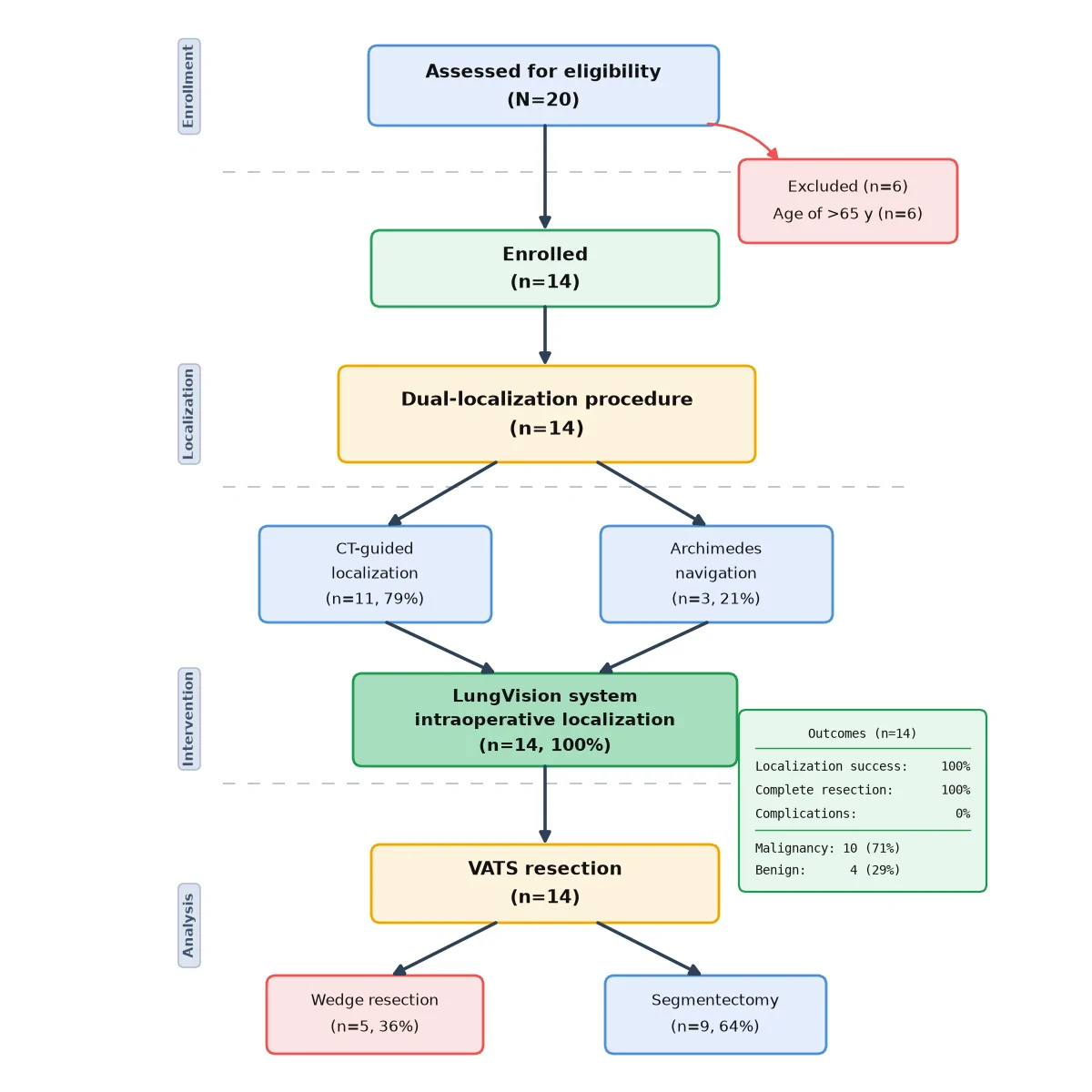
Study flow diagram. Patients assessed for eligibility who exceeded the institutional review board–approved upper age limit (>65 years) were excluded during data verification. The remaining patients underwent dual localization with either a computed tomography (CT)–guided technique or the Archimedes navigation system followed by intraoperative localization using the LungVision system. All patients subsequently underwent video-assisted thoracoscopic surgery (VATS) with either wedge resection or segmentectomy.

The study flow is illustrated in Figure 4. A total of 20 patients were enrolled, of whom 6 (30%) were subsequently excluded during data verification because they exceeded the IRB-approved upper age limit (>65 years), resulting in a final analytic cohort of 14 (70%) patients (Figure 4).

### Patient and Lesion Characteristics

Among the 14 enrolled patients, the average age was 57.2 (SD 9.2; range 28-65) years, with 13 (92.9%) being nonsmokers and 1 (7.1%) being a former smoker. The most common lesion locations were in the left upper lobe (n=5, 35.7%), followed by the right upper lobe, right lower lobe, and left lower lobe (n=3, 21.4% each).

Preoperatively, 85.7% (12/14) of lesions were in the peripheral region, with an average distance of 12.4 (SD 11.8) mm from the visceral pleura. The average tumor size was 9.3 (SD 5.3) mm. All GGOs were smaller than 20 mm, with 78.6% (11/14) measuring no more than 10 mm. Most tumors demonstrated a high GGO ratio, with 92.9% (13/14) exhibiting a GGO ratio of ≥20% (Table 1).

**Table 1:** Characteristics of patients and pulmonary nodules (N=14).

Variable		Values
Sex (male), n (%)		4 (28.6)
Age (y), mean (SD)		57.2 (9.2)
**Smoking history, n (%)**		
	Nonsmoker	13 (92.9)
	Former smoker	1 (7.1)
**Nodule size (mm), mean (SD)**		9.3 (5.3)
	Tumor size≤10 mm, n (%)	11 (78.6)
Tumor depth (from visceral pleura; mm), mean (SD)		12.4 (11.8)
**Tumor location, n (%)**		
	Central	2 (14.3)
	Peripheral	12 (85.7)
	Right upper lobe	3 (21.4)
	Right lower lobe	3 (21.4)
	Left upper lobe	5 (35.7)
	Left lower lobe	3 (21.4)
GGO^a^ ratio≥20%, n (%)		13 (92.9)
GGO ratio, mean (SD)		0.6 (0.4)
Hounsfield units, mean (SD; range)		−320.1 (334.9; −737.3 to 106)

^a^GGO: ground-glass opacity.

### Procedure Outcomes

Using the LungVision system, which integrates real-time fluoroscopy with AI-guided interpretation, all lesions, despite the presence of small and hypodense pulmonary nodules, were accurately targeted. The mean Hounsfield units were −320.1 (SD 334.9), ranging from −737.3 to 106.

Following ICG injection, VATS with wedge resection was performed in 35.7% (5/14) of the patients, whereas 64.3% (9/14) underwent VATS segmentectomy. In all 14 cases, the lesion site indicated by the LungVision-guided tool-in-lesion confirmation was concordant with the preoperative marker location identified intraoperatively. The LungVision-delivered ICG marking was directly visualized on the pleural surface in 78.6% (11/14) of cases.

The mean navigation time, including the entire setup process, was 38.6 (SD 19.5) minutes, and the mean operative time was 95.6 (SD 42.1) minutes. No intraoperative complications or adverse events were observed during navigation or throughout the bronchoscopic procedure. Complete resection was achieved in all cases, with 71.4% (10/14) confirmed as malignant on final pathological examination (Table 2).

**Table 2:** Surgical results (N=14).

Variable				Values
**Surgical procedure, n (%)**				
	VATS^a^ with wedge resection		5 (35.7)	
	VATS with segmentectomy		9 (64.3)	
Operation time (min), mean (SD)				95.6 (42.1)
Navigation time (min), mean (SD)				38.6 (19.5)
**Tumor localization technique^b^, n (%)**				
	**Intraoperative localization**			
		LungVision system	14 (100)	
	**Initial localization method (combined)**			
		CT^c^-guided technique	11 (78.6)	
		Broncus Medical Archimedes system	3 (21.4)	
Dye in appearance (LungVision system), n (%)				11 (78.6)
**Pathological diagnosis, n (%)**				
	Malignancy		10 (71.4)	
	Benign		4 (28.6)	
	Adenocarcinoma		5 (35.7)	
	Minimally invasive adenocarcinoma		3 (21.4)	
	Adenocarcinoma in situ		1 (7.1)	
	Metastatic adenocarcinoma		1 (7.1)	
	Chondroid hamartoma		1 (7.1)	
	Cryptococcosis		1 (7.1)	
	Chronic inflammation		2 (14.3)	

^a^VATS: video-assisted thoracoscopic surgery.

^b^All patients received dual localization: initial localization (computed tomography guided or via the Archimedes system) followed by intraoperative localization using the LungVision system.

^c^CT: computed tomography.

The LungVision system was used with the operating room’s existing C-arm fluoroscopy unit; no additional infrastructure modification or hybrid operating room facility was required for the procedures in this cohort.

## Discussion

### Principal Findings

This study provides preliminary evidence on the clinical feasibility and safety of the LungVision system for intraoperative localization of small, hypodense pulmonary nodules, a category of lesions that poses substantial challenges for thoracoscopic identification. Tool-in-lesion confirmation, the primary outcome, was achieved in all patients in the analytic cohort, complete resection was achieved in all cases, and no localization-related complications occurred. These findings correspond to the feasibility, localization success, and safety objectives stated in the Introduction section. The enrolled cohort was characterized by a high proportion of small and predominantly ground-glass nodules, reflecting a patient population in whom conventional visual and tactile localization is most limited. By integrating preoperative CT datasets with real-time fluoroscopy, the LungVision system uses multimodal image fusion and AI-assisted navigation to enable localization in these technically demanding cases.

### Comparison to Prior Work

The high localization success rate in the present study was consistent with previously published LungVision series reporting 93% to 96.1% success rates [11,12]. Furthermore, no localization-related complications were observed in our study, compared with reported pneumothorax (35%) and dislodgement (2.4%-13%) rates with CT-guided hookwire localization in a previous meta-analysis [10]; however, the small sample size in the present study precludes formal comparison.

Published studies indicate that LungVision reduces effective divergence from the documented 14.5-mm average to approximately 5.9-mm navigation accuracy at the actual target position [12]. While intraoperative cone-beam CT can also address divergence, its routine use is typically restricted to hybrid operating rooms equipped with specialized and costly infrastructure. In contrast, LungVision is compatible with conventional C-arm fluoroscopy systems, which may allow for deployment in settings without dedicated hybrid operating room infrastructure.

C-arm–based tomography, the intraoperative imaging step performed in all cases in this cohort, enables 3D reconstruction of pulmonary lesions through a controlled 60° C-arm rotation with a short acquisition time (15-20 seconds per spin). Previous studies have reported relatively low cumulative radiation exposure associated with LungVision-guided navigational bronchoscopy procedures [13]. Although procedure duration has been reported to vary across different navigation platforms [14,15], direct comparisons across these studies should be interpreted with caution because of differences in procedural settings, patient populations, and study methodologies.

### Implications

From a digital health implementation perspective, LungVision differs from other AI-assisted navigation platforms by relying on software integration with existing fluoroscopic equipment rather than dedicated robotic hardware. Robotic bronchoscopy systems (eg, Ion and MONARCH) typically require specialized equipment and procedural infrastructure. As a result, the implementation requirements of these technologies may differ across institutions. Previous digital health literature has emphasized the role of interoperability and workflow integration in the deployment of new clinical technologies [8,16]. The ability to integrate with standard fluoroscopy systems may facilitate use in health care settings where hybrid operating rooms or robotic platforms are not available. Given regional differences in health care resources, these implementation factors may be relevant when considering the adoption of navigation technologies for pulmonary nodule localization in the Asia-Pacific region.

The LungVision workflow embodies a human-in-the-loop design in which the AI system provides augmented visualization and pathway recommendations, whereas the clinician retains decision-making authority at critical steps—including manual lesion confirmation on the fluoroscopic overlay and final tool-in-lesion verification. This collaborative framework in which AI augments rather than replaces clinical judgment aligns with emerging principles for responsible AI deployment in health care [17]. Future studies should formally evaluate the human-AI interaction dynamics, including operator trust calibration and the impact of AI-generated recommendations on clinical decision-making.

The dual-localization approach used in this study—combining preoperative CT-guided or bronchoscopic marking with intraoperative LungVision navigation—reflects emerging evidence that multimodal strategies optimize surgical accuracy. The VAL-MAP (Virtual-Assisted Lung Mapping) 2.0 multicenter trial [18] demonstrated that tentative staple lines based on surface dye marks alone required modification after visualizing subsurface markers in 50% of cases, underscoring the inadequacy of single-modality localization for deep parenchymal nodules. In the present study, CT-guided methylene blue or Archimedes-guided marking provided initial surface localization, whereas LungVision’s real-time augmented fluoroscopy confirmed tool-in-lesion positioning and guided ICG injection for definitive intraoperative visualization. This complementary approach addresses the fundamental limitation that surface markers alone cannot reliably guide resection depth for nodules embedded within the lung parenchyma.

Beyond surgical localization, the platform’s architecture supports potential future applications, including transbronchial biopsy guidance [19,20], ablative therapy delivery [21,22], and integration with robotic bronchoscopy systems [23,24]. These applications warrant investigation in dedicated clinical trials.

Implementation of this workflow requires familiarity with augmented fluoroscopic imaging and 3D spatial anatomy, as well as coordination among surgeons, anesthesiologists, and imaging personnel. As with other AI-assisted technologies in clinical practice, structured training and multidisciplinary coordination may facilitate implementation and consistent performance [25].

### Limitations

Several limitations should be acknowledged.

First, the mandatory dual-localization design in which all patients underwent both preoperative marking (using either CT-guided or Archimedes-guided techniques) and intraoperative LungVision navigation represents the principal methodological limitation of this study. This approach was adopted to maximize patient safety and reduce interindividual variability through within-subject comparison. The preoperative marker served as a backup to ensure successful surgical localization in all cases. Localization success was defined as tool-in-lesion confirmation under LungVision guidance and was achieved in all patients. However, because all resections were performed with the support of both localization modalities, the final surgical outcome reflects their combined contribution and cannot be attributed exclusively to LungVision. Furthermore, concordance between the preoperative marker and LungVision-guided localization, as well as the incremental clinical value provided by AI-assisted navigation, were not prospectively recorded and, therefore, could not be quantitatively assessed. The absence of a LungVision-only control arm limits the ability to determine the independent effect of the system on localization outcomes. Future controlled studies incorporating a single-modality arm or matched comparison cohort are needed to more rigorously evaluate the incremental value of LungVision.

Second, this was a single-center study with a small sample size, which limits the generalizability of the findings. The IRB-approved protocol for this initial feasibility study also restricted enrollment to patients aged 18 to 65 years, and a substantial proportion of enrolled patients were consequently excluded during data verification. The findings, therefore, may not apply to older patients, who constitute a large share of the lung cancer screening population.

Third, quantitative geometric accuracy measurements—such as the millimeter-scale distance between the AI-guided instrument position and the confirmed lesion centroid—were not prospectively collected. Localization accuracy could therefore be assessed only through procedural end points rather than through direct spatial metrics. Future studies should incorporate prospective geometric accuracy measurement as a primary outcome.

Fourth, this study was neither designed nor powered to characterize a learning curve. An exploratory sequential analysis of navigation time across the consecutive cases was performed and is reported in Multimedia Appendix 2, but the limited number of cases and the heterogeneity of lesion characteristics preclude any conclusion about whether procedural efficiency improved with experience, and the results should be regarded as hypothesis generating only. For the same reason, the influence of operator experience and workflow-related factors on localization outcomes could not be separated from the performance of the system itself.

Fifth, no formal usability assessment (eg, the System Usability Scale) was conducted, so operator experience and interface efficiency were not evaluated, and the independent contribution of the AI algorithm as distinct from operator skill was not quantitatively assessed. These aspects should be addressed in future implementation studies to support evidence-based adoption of this technology [8].

Larger, multicenter studies including broader age ranges are warranted to validate these findings and further define the role of LungVision in thoracic oncology.

### Conclusions

In this single-center pilot study, the LungVision system demonstrated clinical feasibility and a favorable safety profile for intraoperative localization of small, hypodense pulmonary nodules using conventional C-arm fluoroscopy. Tool-in-lesion confirmation was achieved in all cases in the analytic cohort under a dual-localization design. These preliminary findings support further evaluation in larger, prospective multicenter studies across a broader range of thoracic procedures.

## Data Availability

The datasets generated and/or analyzed during this study are not publicly available because they contain patient-level clinical information and are subject to institutional data governance policies. Requests for access to deidentified study data may be submitted to the corresponding author and will be considered on a case-by-case basis subject to applicable ethical, regulatory, and institutional requirements.

## Appendix

Multimedia Appendix 1 Narrated video walkthrough of the LungVision workflow, including system overview and demonstration of intraoperative use in the operating room.

Multimedia Appendix 2 Sequential case plot of intraoperative navigation time.

## Abbreviations

CT: computed tomography
GGO: ground-glass opacity
ICG: indocyanine green
IRB: institutional review board
VAL-MAP: Virtual-Assisted Lung Mapping
VATS: video-assisted thoracoscopic surgery
