# Supplementary material for: Real-Time AI-Augmented Fluoroscopic Navigation for Intraoperative Pulmonary Nodule Localization: Prospective Observational Pilot Study
Source: JMIR Form Res. 2026 Sep 9;10:e94084. doi: 10.2196/94084 (PMC13601874; doi:10.2196/94084)
Supplement: Supplementary file 1 [file formative_v10i1e94084_app2.docx]

**Exploratory sequential analysis of intraoperative navigation time**

**Description of the analysis**

Navigation time, defined as the interval from LungVision system initialization to tool-in-lesion confirmation, was recorded prospectively for each of the 14 consecutive patients in the analytic cohort. To explore whether procedural time changed as experience with the system accumulated, navigation time was plotted against case sequence number, and an ordinary least-squares linear regression line was fitted. This analysis was exploratory and was not prespecified in the study protocol; it was undertaken in response to a reviewer’s suggestion during peer review. No hypothesis test was performed, and no adjustment was made for lesion characteristics or other covariates. The analysis was conducted in Python 3.12 using matplotlib 3.10.7 and seaborn 0.13.2.

**Results**

Mean navigation time across the 14 cases was 38.6 (SD 19.5) minutes, with individual values ranging from 6 to 64 minutes. The fitted regression line had a negative slope (y = −1.13x + 47.0), corresponding to a reduction of approximately 1.1 minutes per successive case, but the coefficient of determination was very low (R² = 0.058), indicating that case sequence accounted for only about 6% of the variance in navigation time. Substantial variability was present between adjacent cases throughout the series.

**Interpretation**

These data are consistent with a modest downward trend in navigation time over successive cases but do not establish a learning curve. With only 14 cases, heterogeneous lesion characteristics, and no adjustment for case complexity, the observed trend is equally compatible with random variation. The result should be regarded as hypothesis-generating and requires confirmation in a larger series designed to assess procedural learning.

**Figure S1.** Sequential case plot of intraoperative navigation time. Each point represents the navigation time in minutes recorded for one of 14 consecutive cases. The dashed line is the ordinary least-squares linear regression trend line (y = −1.13x + 47.0; R² = 0.058).
